# The antimicrobial resistance patterns and associated determinants in *Streptococcus suis *isolated from humans in southern Vietnam, 1997-2008

**DOI:** 10.1186/1471-2334-11-6

**Published:** 2011-01-06

**Authors:** Ngo T Hoa, Tran TB Chieu, Ho DT Nghia, Nguyen TH Mai, Pham H Anh, Marcel Wolbers, Stephen Baker, James I Campbell, Nguyen VV Chau, Tran T Hien, Jeremy Farrar, Constance Schultsz

**Affiliations:** 1 Oxford University Clinical Research Unit, Hospital for Tropical Diseases, Ho Chi Minh City, Vietnam; 2 Pham Ngoc Thach Medical University, Ho Chi Minh City, Vietnam; 3 Hospital for Tropical Diseases, Ho Chi Minh City, Vietnam; 4 Academic Medical Centre - Center for Poverty-related Communicable Diseases, University of Amsterdam, Amsterdam, The Netherlands

## Abstract

**Background:**

*Streptococcus suis *is an emerging zoonotic pathogen and is the leading cause of bacterial meningitis in adults in Vietnam. Systematic data on the antimicrobial susceptibility profiles of *S. suis *strains isolated from human cases are lacking. We studied antimicrobial resistance and associated resistance determinants in *S. suis *isolated from patients with meningitis in southern Vietnam.

**Methods:**

*S. suis *strains isolated between 1997 and 2008 were investigated for their susceptibility to six antimicrobial agents. Strains were screened for the presence and expression of tetracycline and erythromycin resistance determinants and the association of *tet*(M) genes with *Tn*916- like transposons. The localization of tetracycline resistance gene *tet*(L) was determined by pulse field gel electrophoresis and Southern blotting.

**Results:**

We observed a significant increase in resistance to tetracycline and chloramphenicol, which was concurrent with an increase in multi-drug resistance. In tetracycline resistance strains, we identified *tet*(M), *tet*(O), *tet*(W) and *tet*(L) and confirmed their expression. All *tet*(M) genes were associated with a *Tn*916-like transposon. The co-expression of *tet*(L) and other tetracycline resistance gene(s) encoding for ribosomal protection protein(s) was only detected in strains with a minimum inhibitory concentration (MIC) of tetracycline of ≥ 64 mg/L

**Conclusions:**

We demonstrated that multi-drug resistance in *S. suis *causing disease in humans in southern Vietnam has increased over the 11-year period studied. We report the presence and expression of *tet*(L) in *S. suis *strains and our data suggest that co-expression of multiple genes encoding distinct mechanism is required for an MIC ≥ 64 mg/L to tetracycline.

## Background

*Streptococcus suis *is an emerging zoonotic pathogen associated with pigs and can cause severe systemic infections in humans. Up to date approximately 800 human *S. suis *infections have been reported from over twenty countries [1]. The most noticeable incident was a single outbreak in China affecting 215 individuals, of whom 38 died [2]. *S. suis *serotype 2 is the most common serotype associated with human disease [3] and is the most common cause of acute bacterial meningitis in adults in Vietnam [1,4,5].

Antimicrobial susceptibility profiles and the corresponding resistance determinants of *S. suis *have been reported in strains isolated from pigs, but there are only limited data from strains isolated from human patients [6-12]. Resistance to tetracycline and macrolide-lincosamide- streptogramin B (MLS_B_) has been widely reported in *S. suis *strains isolated from pigs in Asia, Europe and North America [8-10,12-15]. In contrast, resistance to chloramphenicol is uncommon [8,14].

The gene *erm*(B), which encodes erythromycin ribosomal methylase, is associated with resistance to MLS_B_, and has been identified in a large number of *S. suis *strains from Hongkong and Belgium [7,9]. Tetracycline resistance in *S. suis *was mainly associated with *tet*(M), *tet*(O), *tet*(W) and *tet*(O/W/32/O); yet, efflux proteins encoded by *tet*(L) and *tet*(K), which also determine tetracyline resistance, have not been reported [7,8,10,11,15-19]. The co-existence of *tet*(M) and *tet*(O) genes in *S. suis *serotype 2 has been detected in limited number of strains isolated from humans and pigs in China and Italy [7,10,11].

We have previously shown that 79/95 (83.2%) *S. suis *serotype 2 strains isolated from adults with meningitis in Vietnam were resistant to tetracycline, and 19/94 (20.2%) were resistant to erythromycin [4]. We have also described the concurrent presence of *tet*(L), *tet*(M), *tet*(O) and *erm*(B) genes in one *S. suis *serotype 2 strain isolated from a Vietnamese patient [20]. However, longitudinal data of antimicrobial resistance over many years and the associated resistance determinants of a large number of *S. suis *strains isolated from human patients have not been reported. Here we describe the antimicrobial resistance pattern, trends and the genetic determinants of tetracycline and erythromycin resistance in *S. suis *strains isolated from adult patients with meningitis in 12 consecutive years (1997-2008), in southern Vietnam.

## Methods

### Bacterial strains

All strains used in this study were obtained from samples which were collected for diagnostic purposes as part of standard care, at The Hospital for Tropical Diseases (HTD) in Ho Chi Minh City, Vietnam. The study protocol was approved by the ethical committee of the HTD (CS/NĐ/09/13). A total of 175 non-duplicate *S. suis *strains, isolated from the cerebral spinal fluid (CSF) of adult patients with meningitis admitted to the HTD between March 1997 and November 2008, were investigated. Bacterial culture, identification and serotyping of *S. suis *was performed as previously described [4]. All strains, with the exception of four strains belong to serotype 14 (n = 3) and serotype 16 (n = 1) [21], were of serotype 2.

### Antimicrobial susceptibility testing

Antimicrobial susceptibility testing was performed by assessing the minimum inhibitory concentration (MIC) for all isolates using E-test (AB Biodisk, Sweden) according to the manufacturer recommendations. The antimicrobials tested were penicillin, ceftriaxone, vancomycin, chloramphenicol, erythromycin and tetracycline. Antimicrobial resistance was assessed using breakpoints recommended by the Clinical and Laboratory Standard Institute (CLSI) guidelines 2008 (M100-S18). There are currently no breakpoints recommended for *S. suis*, therefore, breakpoints for viridans group *Streptococci *were used (Table 1). *Streptococcus pneumoniae *strain ATCC 49619 was used for quality control purpose. Erythromycin resistance phenotypes were identified using the triple disk diffusion test as described [22].

**Table 1:** Distribution of minimal inhibitory concentrations of antimicrobial agents tested in Streptococcus suis strains isolated from patients with meningitis

Antimicrobial Agent		Minimal Inhibitory Concentrations			
	Breakpoint* (mg/L)	**Range (mg/L)**	**MIC_50_^# ^(mg/L)**	**MIC_90_^# ^(mg/L)**	**Resistant strains (%)**

Tetracycline	S ≤ 2; R ≥ 8	0.125-->256	24	32	88.6

Erythromycin	S ≤ 0.25; R ≥ 1	0.016-- >256	0.064	>256	22.2

Chloramphenicol	S ≤ 4; R ≥ 16	0.5-96	3	4	8.6^@^

Penicillin	S ≤ 0.12; R ≥ 4	0.012-0.064	0.032	0.047	0

Ceftriaxone	S ≤ 0.5	0.025-0.38	0.094	0.125	0
Vancomycin	S ≤ 1	0.25-0.5	0.38	0.5	0

*: Breakpoints are based on equivalent CLSI breakpoints for *Streptococcus spp*. viridans group

^#^: MIC_50_, MIC_90 _are the MIC values inhibiting growth of 50% and 90% of tested isolates, respectively.

^@^: Including the intermediate resistant isolates

### Detection of erythromycin and tetracycline resistance determinants

Genomic DNA was extracted using DNEasy tissue kit (Qiagen, United Kingdom). The *erm*(A), *erm*(B), *mef*(A), *tet*(M), *tet*(O), *tet*(L) and *tet*(K) genes were detected by multiplex PCR as previously described [23]. The presence of *tet*(W) and mosaic gene *tet*(O/W/32/O) was screened using primers as described in Table 2. DNA sequencing of PCR amplicons was performed to confirm the presence of the full length *tet*(W) gene using primers tet(W)-F, tetO-2-F, tetO-2-R and tet(W)-R (Table 2).

**Table 2:** Primers used to amplify the tetracycline and macrolide resistance determinants and for sequencing purpose

Primer	Sequence (5' - 3')	Product size	Position in coding sequence	Reference
erm(A)-F	CCCGAAAAATACGCAAAATTTCAT	590	15-38	[23]
erm(A)-R	CCCTGTTTACCCATTTATAAACG		604-582	
erm(B)-F	TGGTATTCCAAATGCGTAATG	745	203-183	
erm(B)-R	CTGTGGTATGGCGGGTAAGT		541-522	
mef(A)-F*	CAATATGGGCAGGGCAAG	317	38-55	
mef(A)-R*	AAGCTGTTCCAATGCTACGG		352-333	
tet(M)-F	GTGGACAAAGGTACAACGAG	406	106-125	
tet(M)-R	CGGTAAAGTTCGTCACACAC		511-492	
tet(O)- F	AACTTAGGCATTCTGGCTCAC	515	13-43	
tet(O)- R	TCCCACTGTTCCATATCGTCA		527-507	
tet(K)-F	GATCAATTGTAGCTTTAGGTGAAGG	155	344-368	
tet(K)-R	TTTTGTTGATTTACCAGGTACCATT		498-474	
tet(L)-F	TGGTGGAATGATAGCCCATT	229	384-403	
tet(L)-R	CAGGAATGACAGCACGCTAA		612-593	
16S-rDNA-F	GAGTACGACCGCAAGGTTGA	100	886-905	
16S-rDNA -R	CTGGTAAGGTTCTTCGCGTTG		985-964	

ss-Tn*916*-1	GCCATGACCTATCTTATA	476	16083-16100	[24]
ss-Tn*916*-2	CTAGATTGCGTCCAA		16559-16545	

tet(32)For	GAACCAGATGCTGCTCTT	620	619-637	[28]
Tet(32)Rev	CATAGCCACGCCCACATGAT		1239-1220	

SScps2J-F	CAAACGCAAGGAATTACGGTATC	236	209-231	[29,30]
SScps2J-R	CATTTCCTAAGTCTCGCACC		445-426	

tet(L)Ng-F	TCGTTAGCGTGCTGTCATTC	698	680-700	[31]
tet(L)-R-pDG364	CTTAGAAATCCCTTTGAGAAT		1378-1358	This study

tet(W)- F	TTGGAATTCTTGCCCATGTAGACGC	1872	18-42	This study
tet(W)- R	TTGTCCAGGCGGTTGTTTGGAC		1889-1868	
tet(W)-F-HN	GGTGCAGTTGGAGGTTGTTT	410	1179-1198	
tet(W)-R-HN	CCTTCAATGCCTGTTCCAAT		1569-1588	
tet(O)-F-pDG364	ATGAAAATAATTAACTTAGG	1920	1-19	
tet(O)-R-pDG364	TTAAGCTAACTTGTGGAACA		1920-1901	
ss-tet(M)-whole-F	ACAGACAAAGAACTATCCTTAATG	2500	419-396	
ss-tet(M)-whole-R	GTACCCAGTTTAAGAATACCTTTATC		161-136	

**Additional primers used for sequencing**				

tet(O)-2-F	TTCAAGACGCCTCCCTGTTC		679-698	This study
tet(O)-2-R	ATTTGGCGGGACTTCTATGTGG		1318-1296	
ss-tet(M)-Fseq1	GTTAAATCACTACGATAT		1763-1745	
ss-tet(M)-Rseq1	ATAGTGTTCTTGGAGATA		906-929	
ss-tet(M)-Fseq2	GTATAATTTCATGTGTCG		1162-1144	
ss-tet(M)-Rseq2	AGATGGCGTACAAGCACA		305-323	
ss-PAI-P8-tet(M)-R	GCCCTTTTGGGTTTTTGAAT		-33- -14	
ss-PAItetM-P9over F	GGGAATCCCCATTTTCCTAA		366-347	

*: These primers were named mef(A/E) in the referenced publication.

*S. suis *strain BM407, which contains the genes *tet*(M), *tet*(O), *tet*(L) and *erm*(B), and *S. suis *strain BM331 containing the full length *tet*(W) gene, were used as positive control strains [20]. The presence of *Tn916*-like conjugative transposons was screened for in all *tet*(M) positive strains by amplification of the x*is-Tn *gene of *Tn916*, as described elsewhere [24]. The association of the *tet*(M) gene with *Tn916 *was demonstrated in *xis-Tn *and *tet*(M) positive strains by an additional PCR using primers Tn916-2 and P8-tet(M)-R (Table 2).

### Detection of expression of tetracycline resistance genes

The expression of tetracycline resistance genes was determined in all *S. suis *strains possessing multiple tetracycline resistance determinants according to techniques described previously with modification [17]. A tetracycline disk (30 ug) was placed on a Mueller-Hinton agar plate inoculated with a 0.5 McFarland suspension of the test *S. suis *strain. Colonies surrounding the zone of inhibition were harvested for RNA extraction. cDNA was synthesized using random hexamer primers and the subsequent products were used as templates for multiplex PCR for detection of tetracycline resistance genes *tet*(M), *tet*(O), *tet*(L), and *tet*(K) (Table 1). RT-PCR of *cps2J *or *16SrRNA *gene products was used as internal control.

### Pulse Field Gel Electrophoresis (PFGE) and Southern blot to detect the location of *tet*(L) in *S. suis *isolates

Agarose plugs were prepared and solidified plugs were processed as described [25]. *SmaI *digested genomic DNA was electrophoresed by PFGE in a CHEF mapper (BioRad, Vietnam). The separation of plasmid DNA and chromosomal DNA was achieved using the conditions suitable for separation of DNA fragments between 1.2 Kb and 100 Kb. *S. suis *strain BM407, which was shown previously to contain one plasmid and the *tet*(L) gene, located on the chromosome, was used as positive control [20]. Ten nanograms (10 ng) of PCR amplicon *tet*(L) (698 bp) was included as a positive control for Southern blotting in each gel. DNA was transfered to the nitrocellulose membranes using a vacuum blotter (BioRad, Vietnam).

The probe for detection of the *tet*(L) gene after Southern blotting was prepared using the same PCR amplicon, which was labeled using the ECL direct nucleic acid labeling and detection kit (GE Healthcare, HCMC, Vietnam).

### Statistical analysis

We tested for a linear time trend in differential antimicrobial resistance rates using logistic regression with the antimicrobial susceptibility of the strain as the outcome and the calendar year of the strain collection as the covariate. As only one strain was collected in 1997, it was excluded from all statistical analyses. Strains with intermediate resistance were considered resistant for the purpose of these analyses. The MIC of tetracycline was compared between tetracycline resistant *S. suis *groups. Four groups of tetracycline resistant strains were defined as follows: strains carrying *tet*(M); strains carrying *tet*(O); strains carrying *tet*(M) and *tet*(O); and strains carrying *tet*(M) and *tet*(L) or *tet*(M), *tet*(O) and *tet*(L). The sole strain which contained *tet*(M) and *tet*(L) was added to the latter group prior to the analysis. We used a Kruskal-Wallis test for the overall comparison of the four groups and then performed pair-wise comparisons using Wilcoxon rank-sum tests with Holm correction for multiple testing. All reported p-values correspond to two-sided tests and analyses were performed with R 2.8.1 (R Foundation for Statistical Computing, Vienna, Austria).

## Results

### Antimicrobial susceptibility profiles and macrolide resistance phenotype

**Figure 1:**
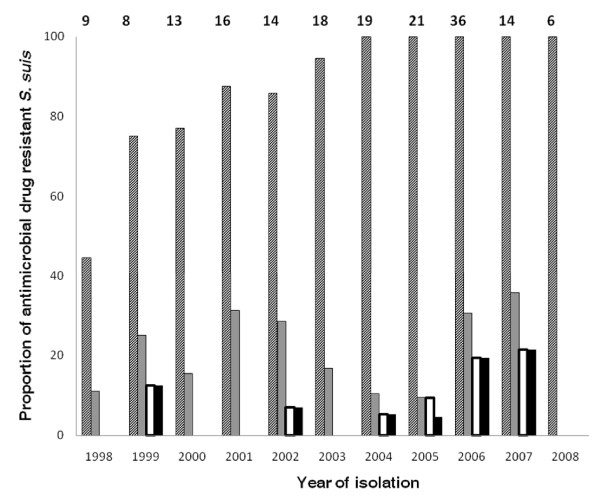
**Proportional distribution of antimicrobial resistant *S. suis *strains between 1998 and 2008**. *Streptoccoccus suis *strains were isolated from patients with acute bacterial meningitis admitted to the Hospital for Tropical Diseases between 1998 and 2008. The numbers above each column correspond to the total number of strains isolated in each year (the lone isolate from 1997 is not included. Diagonal, grey and white bars represent the proportion of strains resistant to tetracycline, erythromycin and chloramphenicol, respectively. Black bars represent the proportion of MDR strains (resistant to tetracycline, erythromycin and chloramphenicol).

All 175 *S. suis *strains were fully susceptible to penicillin, ceftriaxone and vancomycin (Table 1). Fourteen strains were sensitive to all 6 antibiotics tested. A total of 159/175 (90.9%) strains were resistant to tetracycline and 39/175 (22.2%) strains were resistant to erythromycin. Fifteen (8.6%) strains were resistant to chloramphenicol, including those demonstrating intermediate resistance (Figure 1). The macrolide resistance phenotype was identified using triple-disk tests in 38/39 erythromycin resistant strains. Of these, 37 (97.4%) strains demonstrated a cMLS_B _phenotype and the remaining strain expressed the iMLS_B _-A phenotype.

Resistance to tetracycline significantly increased over time (OR = 1.77 [95% CI 1.36-2.30] per year, p < 0.001). The proportion of *S. suis *strains demonstrating intermediate or full resistance to tetracycline was 80.8% (61/78 strains) in the period 1998-2003 and 99% (95/96 strains) in the period 2004-2008 (Figure 1). A significant increase was also observed in chloramphenicol resistance (OR = 1.36 [95% CI 1.05-1.76] per year, p = 0.02) which rose from 2.5% (2/78 strains) in 1998-2003 to 13% (12/96 strains) in 2004-2008. In contrast, there was no significant increase in erythromycin resistance observed over 11 years (OR = 1.04 (95% CI 0.91-1.19) per year; p = 0.56) and the proportion of erythromycin resistant strains was the same (22%) in both periods of time (Figure 1). Multi-drug resistance (MDR), defined as resistance to tetracycline, erythromycin and chloramphenicol, significantly increased over time (OR = 1.36 [95% CI = 1.04-1.77] per year, p = 0.02). Two (2.5%) and 12 MDR strains (12.5%) were isolated in 1998-2003 and in 2004-2008, respectively (Figure 1).

### Tetracycline and erythromycin resistance determinants

We screened for the presence of tetracycline and erythromycin resistance determinants in 169 isolates, including two *S. suis *serotype 14 and one *S. suis *serotype 16 strains. The tetracycline resistance determinant *tet*(M) was successfully amplified in 129/153 (84.3%) tetracycline resistant isolates. The presence of the *xis*-*Tn *gene of *Tn916*-like transposons and its association with *tet*(M) was further screened by PCRs in strains that contained *tet*(M). All *tet*(M) positive *S. suis *strains produced amplicons for both the *xis*-*Tn *gene and the DNA fragments linking *xis*-*Tn *and *tet*(M) genes. The *tet*(O) gene was found in 33 (21.6%) strains and *tet*(L) was detected in 5 (3.3%) strains. The three genes *tet*(M), *tet*(O) and *tet*(L) were concomitantly amplified in 4 (2.6%) strains. We detected several different combinations of tetracycline resistance genes in individual strains. The genes *tet*(M) and *tet*(L) or *tet*(M) and *tet*(O) were found in 6 (3.9%) isolates, whilst single tetracycline resistance gene *tet*(M) or *tet*(O) were present in 139 (90.8%) strains. We identified 2/92 tested strains containing the full-length *tet*(W) gene.

Resistance determinant *erm*(B) was detected in 36/38 (94.7%) erythromycin resistant strains. All possible combinations of the *erm*(B) gene with tetracycline resistance genes *tet*(M), *tet *(O) and *tet*(L) were found. We were unable to amplify amplicons of the genes *tet*(K), *tet*(O/W/32/O), *erm*(A) and *mef*(A) in any of the strains studied. Statistical analysis showed no association between tetracycline and erythromycin resistance of *S. suis *strains (p = 0.53).

### Defining the genomic location of *tet*(L) in *S. suis*

**Figure 2:**
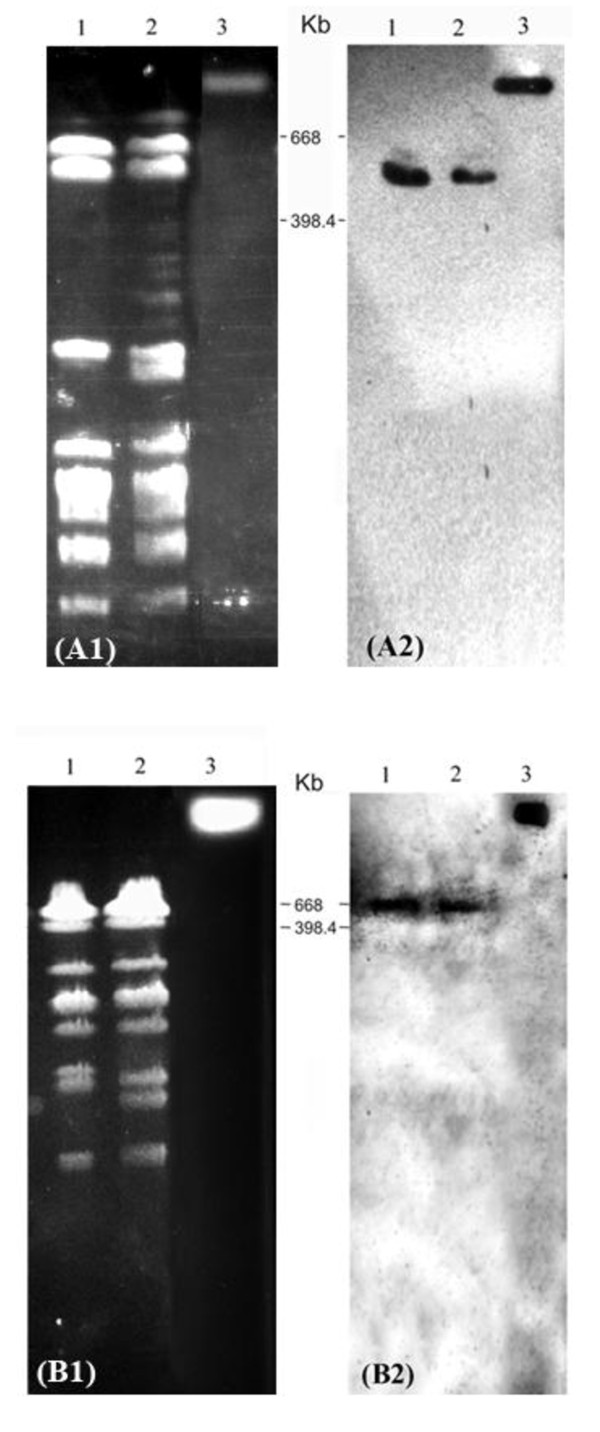
**Chromosomal location of *tet*(L) genes in *S. suis *isolates**. (A1, B1) PFGE patterns of *Sma*I digested genomic DNA of four *S. suis *serotype 2 strains, (A2, B2): Southern blot hybridization of *SmaI *digested genomic DNA of four strains with 698 bp- *tet*(L) probe. (A) Lane 1: *S. suis *BM308; lane 2: *S. suis *BM407; (B) lane 1: *S. suis *EN031; lane 2: *S. suis *EN241. (A, B) Lane 3: 698 bp-*tet*(L) PCR amplicons, which was added to the well of PFGE gel one hour prior to completion of the PFGE running programme. Molecular weights of marker bands, consisting of *Xba*I digested genomic DNA of *Salmonella *serotype Braenderup strain (H9812), spanning the region containing the positive signals of *tet*(L), are indicated.

The gene *tet*(L) was previously found on a conjugative element integrated into the chromosome of *S. suis *strain BM407 [20]. However, *tet*(L) is often found on small transmissible plasmids [26]. Using PFGE and Southern blotting we investigated whether *tet*(L) genes of the other *tet*(L) PCR positive *S. suis *strains were also located on the chromosomes or on replicons additional to chromosomes. Figure 2 shows hybridization patterns for the presence of *tet*(L) after Southern blotting of *Sma*I digested DNA of four *S. suis *strains BM308, BM407, EN031 and EN241. Only strain BM407 was found to contain plasmid DNA (data not shown), which confirms a previous observation that this strain contains a 24,579 bp-pBM407 plasmid with unknown function [20]. Southern hybridization confirmed the location of *tet*(L) on fragments between 398 and 668 Kb in size on the chromosomes of studied strains.

### Expression of tetracycline resistance determinants

The MICs of tetracycline differed significantly between the 4 groups of strains containing tetracycline resistance genes (p < 0.001, Kruskal-Wallis test). Pair-wise comparisons revealed that the MICs to tetracycline for strains containing *tet*(M) and *tet*(L) or *tet*(M), *tet*(O) and *tet*(L) genes was higher than for strains possessing both *tet*(M) and *tet*(O) genes, and for strains containing only *tet*(M) or *tet*(O) (Table 3).

**Table 3:** Groups of S. suis strains with different combinations of tetracycline genes and respective minimal inhibitory concentrations.

Number of isolates	Group	Tetracycline resistance genes	MIC_50_* (mg/L)	Range of MIC (mg/L)	p-values^#^
5	1	*tet*(M), *tet*(O) and *tet*(L) or *tet*(M) and *tet*(L)	64	64 - 256	0.01 (group 1 vs. 2)
					<0.001 (group 1 vs. 3)
					0.002 (group 1 vs. 4)
					0.002 (group 2 vs. 3)
					0.03 (group 2 vs. 4)
					0.08 (group 3 vs. 4)

8	2	*tet*(M) and *tet*(O)	32	24 - 48	

116	3	*tet*(M)	24	3 - 48	

22	4	*tet*(O)	24	16 - 32	

* MIC_50 _is the MIC value inhibiting growth of 50% of tested isolates

^**# **^Pair-wise comparisons based on Wilcoxon rank-sum tests with Holm correction for multiple testing

Among the four *S. suis *strains (BM308, BM407, EN031 and EN241) that possessed the three tetracycline resistance genes *tet*(M), *tet*(O), and *tet*(L), only strain BM407 had a MIC to tetracycline ≥256 mg/L, whilst the MIC was 64 mg/L for the other three strains. We attempted to confirm the co-transcription of the three tetracycline resistance determinants in these four strains. The transcription of all 3 genes simultaneously was only detected in strain BM407. We could not detect the expression of *tet*(M) in one strain and mRNA from *tet*(O) was not detected in the other two strains (data not shown). However, we detected the presence of mRNAs from *tet*(M) and *tet*(L) in the single strain which contained both these determinants, which had a MIC of 64 mg/L to tetracycline.

## Discussion

Invasive human infections by *S. suis *are becoming increasingly reported in Asia. We have observed an increase in the number of infections caused by *S. suis *serotype 2 at the Hospital for Tropical Diseases in HCMC since 1998. This increase could be due to increased awareness and/or a true increase in the incidence of disease. Here we have shown an increase in a tetracycline and chloramphenicol resistance over an eleven- year period. This suggests that continuous surveillance for antimicrobial resistance in *S. suis *is important to guide current and future treatment of human and porcine disease caused by *S. suis*.

Our data show that all *S. suis *strains isolated from adult patients over a twelve year period in Vietnam were sensitive to penicillin. Whilst *S. suis *resistant to penicillin has been reported in isolates from pigs in Denmark, Poland and Portugal [8], it does not appear to be emerging in Vietnam.

We did, however, demonstrate a high proportion of tetracycline resistance (91%). Tetracycline resistance has been reported previously in *S. suis *isolated from pig in various location in Asia and Europe [7,10-12]. Since *S. suis *infection in human is associated with exposure to pigs or contaminated pork [1], the increase in tetracycline resistance in strains isolated from humans may be related to tetracycline usage in animal production for prophylaxis or therapy leading to positive selection of resistant strains. Although data on antimicrobial drug use in animal husbandry in Vietnam is currently unavailable, we observed a tetracycline resistance rate of 100% in 45 *S. suis *serotype 2 strains isolated from a representative sample of healthy slaughterhouse pigs in southern Vietnam in 2006 and 2007, suggesting that current tetracycline resistance rates in pig carriage strains are extremely high (Hoa *et al*, in preparation).

Resistance to tetracycline was encoded by multiple determinants including *tet*(L) in this strain collection. We were able to demonstrate that the *tet*(L) gene was located on the chromosomes of four strains and the *tet*(L) transcription was detected in all five *S. suis *strains with *tet*(L) amplicons positive. The presence of the gene *tet*(L) has, until now, only been described in the genome sequence of *S. suis *serotype 2 strain BM407, which was isolated from a patient with meningitis in Vietnam [20]. The most frequently identified tetracycline resistance gene was *tet*(M) and its association with the presence of *Tn916 *like elements was confirmed in all *tet*(M) positive strains. Mobile genetic elements, including transposons and integrative elements, are common in *Streptococci *such as *S. suis *[11]. The colonization of *S. suis *in the upper respiratory and gastrointestinal tracts of healthy pigs is well known, and the transfer of mobile DNA in such locations is common [20].

In our study, strains carrying multiple tetracycline resistance genes were likely to associate with higher MIC to tetracycline than those carrying single or two resistance genes. The difference in MIC to tetracycline in four strains, all harbouring *tet*(M), *tet*(O), and *tet*(L), was likely due to the simultaneous expression of these genes. The proteins encoded by *tet*(M) and *tet*(O) prevent tetracycline binding to its target on the bacterial ribosome, whilst *tet*(L) encodes a membrane-associated protein, which facilitates tetracycline export from the cell [27]. We surmise that the simultaneous expression of tetracycline resistance genes encoding for efflux protein and ribosomal protection proteins in the same *S. suis *strain results in an elevated MIC to tetracycline.

Erythromycin resistance has been reported in *S. suis *strains isolated from human infections in Hong Kong, with a proportion of resistant strains similar to that observed in our study (21.2%) [7]. The proportions of resistance to erythromycin of *S. suis *strains isolated from pig are higher in other countries including Denmark (29.1%), the United Kingdom (36%), the Netherlands (35%) and Poland (30.6%), France (64.6%), China (67.2%), Portugal (75%) and Italy (78%) [8,10,12]. We were unable to amplify target sequences for the genes *erm*(A), *erm*(B) and *mef*(A) in two erythromycin resistant strains. These data suggest that other erythromycin resistance determinants may be present in *S. suis *and requires additional investigations.

The usage of chloramphenicol in agriculture has been banned in Vietnam since 2003. However, other amphenicols (such as florfenicol) are still allowed to use in agriculture and animal husbandry in Vietnam. The increasing proportion of chloramphenicol resistant *S. suis *strains isolated from humans in Vietnam may be associated with other amphenicols use. It is also possible that the chloramphenicol resistance determinants are under co-selection in strains that are resistant to other antimicrobials currently approved for veterinary use in the prevention and treatment of infections, such as tetracyclines and macrolides. It is noteworthy that all chloramphenicol resistant strains in our study were additionally resistant to tetracycline or erythromycin or both. The genome sequence publication of *S. suis *strain BM407 described that the resistance determinants *erm*(B), *tet*(O), *cat, tet*(L) and *tet*(M) are all located within a 40 Kb DNA region of a conjugative mobile element in this strain [20]. This would support the hypothesis of co-selection of antimicrobial resistance determinants.

## Conclusion

This study reported an increasing proportion of antimicrobial resistant *S. suis *isolates from adult patients with meningitis in southern Vietnam over 11 consecutive years. We demonstrated the presence and expression of *tet*(L) gene in *S. suis*. Our results imply that the simultaneous expression of the *tet*(L) gene and additional tetracycline resistance gene(s), encoding for the ribosomal protection protein(s), in *S. suis *is associated with higher MIC to tetracycline.

## Competing interests

The authors declare that they have no competing interests.

## Authors' contributions

NTH designed the experiments, performed data analysis, interpretation and wrote the first draft of the manuscript. TTBC and PHA performed experiments and data analysis. HDTN and NTHM were involved in study design and clinical sample collection. MW performed statistical analysis and contributed in writing the manuscript. JC and SB contributed to data collection and writing of the manuscript. NVVC and TTH involved in study design and data collection. JF and CS contributed to study design, interpretation, coordination, and writing of the manuscript. All authors read and approved the final version of the manuscript.

## Author's information

NTH is a microbiologist at OUCRU-HTD HCMC, Wellcome Trust Oversea Programme in Vietnam. Her main research interest is emerging zoonotic pathogens with the current focus on *S. suis *infection in humans and pigs.

## Pre-publication history

The pre-publication history for this paper can be accessed here:

http://www.biomedcentral.com/1471-2334/11/6/prepub
